# Multiplexed MRM-based protein quantification of putative prognostic biomarkers for chronic kidney disease progression in plasma

**DOI:** 10.1038/s41598-020-61496-z

**Published:** 2020-03-16

**Authors:** Manousos Makridakis, Georgia Kontostathi, Eleni Petra, Rafael Stroggilos, Vasiliki Lygirou, Szymon Filip, Flore Duranton, Harald Mischak, Angel Argiles, Jerome Zoidakis, Antonia Vlahou

**Affiliations:** 1 Biotechnology Division, Biomedical Research Foundation, Academy of Athens (BRFAA), Athens, Greece; 2 RD-Néphrologie, Montpellier, France; 3 Mosaiques Diagnostics, Hannover, Germany

**Keywords:** Proteomic analysis, Prognostic markers

## Abstract

Current diagnostic measures for Chronic Kidney Disease (CKD) include detection of reduced estimated glomerular filtration rate (eGFR) and albuminuria, which have suboptimal accuracies in predicting disease progression. The disease complexity and heterogeneity underscore the need for multiplex quantification of different markers. The goal of this study was to determine the association of six previously reported CKD-associated plasma proteins [B2M (Beta-2-microglobulin), SERPINF1 (Pigment epithelium-derived factor), AMBP (Protein AMBP), LYZ (Lysozyme C), HBB (Hemoglobin subunit beta) and IGHA1 (Immunoglobulin heavy constant alpha 1)], as measured in a multiplex format, with kidney function, and outcome. Antibody-free, multiple reaction monitoring mass spectrometry (MRM) assays were developed, characterized for their analytical performance, and used for the analysis of 72 plasma samples from a patient cohort with longitudinal follow-up. The MRM significantly correlated (Rho = 0.5–0.9) with results from respective ELISA. Five proteins [AMBP, B2M, LYZ, HBB and SERPINF1] were significantly associated with eGFR, with the three former also associated with unfavorable outcome. The combination of these markers provided stronger associations with outcome (p < 0.0001) compared to individual markers. Collectively, our study describes a multiplex assay for absolute quantification and verification analysis of previously described putative CKD prognostic markers, laying the groundwork for further use in prospective validation studies.

## Introduction

Chronic kidney disease (CKD), defined as reduced kidney function and/or evidence of kidney damage, is a major public health problem throughout the world. Major health problems around the globe, with consistent prevalence rates of 10–13% have been reported (depending on reference group and stage)^1,2^. Disease management is characterized by excessive financial costs (with expenses associated with CKD treatment, exceeding 100 B € in total, annually in Europe)^2,3^. Common risk factors for CKD include ageing of the population and increased rates of diabetes and hypertension^2,4^. Of note, patients with CKD have an overall 30-fold increased risk for suffering from Cardio Vascular Disease (CVD) complications, this being the main cause of CKD-associated deaths. Specifically, ~45% of patients with CKD stage 4–5 die from CVD^5^ and risk of CVD increases with CKD severity, which is already significantly higher in early CKD compared to non-CKD^6,7^. Early identification of CKD and addressing modifiable risk factors is recommended, as it can reduce the risk of kidney failure and CVD by up to 50%^8^. Early detection or prediction of complications enable early intervention, thus could increase the chances for higher treatment efficacy^9–11^.

CKD diagnosis is currently based on the detection of reduced estimated glomerular filtration rate (eGFR) and/or albuminuria, as indicators of renal dysfunction^12,13^. However, these markers have substantial limitations in evaluating CKD progression. Albuminuria quantification and categorization into normalbuminuria (<30 mg/24 hours), microalbuminuria (30–300 mg/24 hours) and macroalbuminuria (>300 mg/24 hours)^13,14^ is commonly used alone or in combination with eGFR to predict kidney damage^15^. However, a significant proportion of patients progress without exhibiting significantly increased urinary albumin levels^16^. Collectively, there is ample room for improvement especially with respect to prognosis of CKD complications, in part due to the fact that the main biomarkers currently used (albuminuria, eGFR) are neither an early indicator nor linked to molecular pathophysiology involved in disease progression, but rather the functional consequences of the already settled pathological modifications.

More accurate evaluation of progression risk could be of significant benefit, since treatment options are presently available^9–11^. As such, biomarker research in the field has been rigorous with ample biomarker data collected until now^17–20^. Various plasma proteins have been associated with CKD progression, with CVD, and with patient-relevant outcome [End Stage Renal Disease (ESRD), death]. These include beta-2 microglobulin (B2M), neutrophil gelatinase-associated lipocalin (NGAL), kidney injury molecule-1, liver-type fatty acid binding protein, cystatin-C, FGF23, as prominent examples^21–25^.

Even though associations with outcome have been reported, the overall suboptimal accuracies of individual markers emphasize the need to establish panels or multi-parametric classifiers, better reflective of the substantial phenotypic and molecular heterogeneity of CKD. Along these lines, a urinary peptide classifier, CKD273, consisting of 273 peptides, fragments of multiple kidney-specific as well as plasma proteins detected by the use of capillary electrophoresis (CE) in combination to mass spectrometry (MS) has been developed, subsequently evaluated^26–28^ and received a letter of support by the US Food and Drug Administration (FDA, USA) for use in early detection of nephropathy in diabetic patients^29^. CKD273 has been found to predict progression at early CKD stages (eGFR > 70 mL/min/1.73 m^2^) more accurately than albuminuria^30^, and has been applied in the proteome-guided intervention trial, PRIORITY^31^.

Prediction of progression to ESRD or CVD-related endpoints is critical for disease management^26^. As a step in this direction, we aimed at establishing absolute quantification assays and performing an initial evaluation of the prognostic value of six plasma proteins, when measured in a multiplex format, for a patient-relevant endpoint, death. The employed method is the targeted mass spectrometry-based multiple reaction monitoring (MRM), relying on the quantification of one or more unique/proteotypic peptides corresponding to a target protein^32,33^ without using antibodies^33^. The applied quantification strategy is the Absolute Quantification (AQUA) workflow^34^, involving stable isotope-labeled peptides, spiked into the sample of interest at predefined amounts and measured simultaneously with the respective endogenous peptides^35,36^. The selection of the specific markers was guided by the existence of mass spectrometry-based data for these proteins^37^, their expected abundance levels based on the existent literature, targeting to avoid extensive pre-fractionation, as well as levels of evidence for the association of these proteins with unfavorable outcome. Using the study by Glorieux *et al*.^37^ as a basis, involving high resolution LC-MS/MS analysis of plasma proteome from patients with CKD and reporting on associations to outcome, in combination to the aforementioned criteria, the tested panel includes B2M^38–46^ and SERPINF1^47–50^, having been largely studied in CKD and serving as positive controls for the approach, as well as AMBP, LYZ, HBB, and IGHA1 with some earlier reported associations^37^, nevertheless not been validated yet in association with disease progression via absolute quantification.

## Results

### Candidate biomarkers and assay establishment

The markers to be quantified were selected from the list of differentially expressed proteins in plasma of haemodialysis (HD) patients with CKD stage 5 versus CKD stages 2–3 in Glorieux *et al*.^37^, further considering availability of proteotypic peptides for MS quantification assays, as described in Methods. To avoid extensive pre-fractionation steps potentially compromising assay reproducibility and applicability, only markers with expected relatively high (>100 ng/ml) plasma abundance levels reported in the literature and existing proteomic databases were considered **(**Supplementary Table S1**)**. The selected marker candidates were B2M, SERPINF1, AMBP, LYZ, HBB, and IGHA1. Following a development phase, involving testing multiple peptides per marker, as described in Methods, standard curves per selected peptide were generated to ensure reproducibility, minimal or lack of matrix effects and linearity in measurements in the range of expected quantified values. Standard curves using synthetic isotope labeled peptide standards (SIS), specific per marker, were generated using a pooled plasma sample (from patients at CKD stages 2–5, n = 4), as matrix.

**Figure 1:**
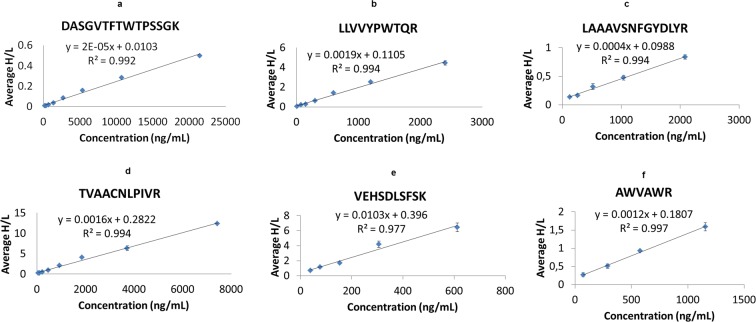
Standard curves generated using labeled peptide standards per marker spiked in plasma matrix at different concentrations. The peptides specifically correspond to: (**a**) DASGVTFTWTPSSGK (IGHA1), (**b**) LLVVYPWTQR (HBB), (**c**) LAAAVSNFGYDLYR (SERPINF1), (**d**) ΤVAACNLPIVR (AMBP), (**e**) VEHSDLSFSK (B2M), (**f**) AWVAWR (LYZ). For each dilution point n = 3 replicates were analyzed.

As shown in Fig. 1, in all cases, R^2^ coefficient of at least 0.992 and for a minimum 4-point concentration ranges, spanning the expected abundances were observed. In addition, CVs of the ratio of the abundance of the standard (SIS) to the endogenous (NAT; SIS/NAT) peptide for all dilution points ranged from 0.40–16.06%, (only exception was for the concentration 72.18 ng/mL of LYZ peptide with CV of 20.79%) suggesting good reproducibility and minimal matrix effects for the assays. The LLOQ (Lower Limit of Quantification) ranged from 9.38–167.5 ng/mL, defined as the lowest qualified concentration level of SIS peptide corresponding to SIS/NAT ratio with CV < 20%^51–53^ (with sole exception the LLOQ of LYZ corresponding to SIS/NAT ratio with CV = 20.79%-marginally higher than CV = 20%). Consequently, the LLOD (Lower Limit of Detection) for the peptides was estimated at 6.54–112.52 ng/mL, based on the following formula 3.3*sd/slope: (https://www.ich.org/). (Supplementary Table S2**)**.

### Differential expression analysis for baseline data

Using the established assays, quantification of the selected markers was performed on all baseline samples from 72 patients that were included in the original study. These included patients with CKD stage 5 (n = 33), CKD stage 4 (n = 21), CKD stage 3 (n = 12), and CKD stage 2 (n = 6) at baseline. Thirty-five events (deaths) were observed (mean follow-up time 3.61 ± 2.96 years), with n = 34 patients being alive at the last contact date (mean follow-up time 5.21 ± 2.77 years). For 3 patients, no follow-up information was available. The main clinical data are summarized in Table 1 and more details can be found in Supplementary Table S3.

**Table 1:** Patient cohort: The table summarizes the demographic and clinical data of the patients included in the study, as per availability.

N	CKD groups			
	CKD5*	CKD4	CKD3	CKD2
	33 (24 HD)	21	12	6
Male/Female	24/9	11/10	7/5	3/3
eGFR (EPI)	*11.14 ± 2.28	22.51 ± 4.71	42.19 ± 9.22	68.46 ± 6.77
Age (years)	73.30 ± 12.30	74.80 ± 5.79	70.58 ± 13.54	54.33 ± 12.51
Serum creatinine [mg/dL]	6.17 ± 2.10	2.46 ± 0.48	1.53 ± 0.32	1.07 ± 0.11
**Aetiologies**				
Diabetes	16	13	5	2
**Other than Diabetes	17	8	7	4
N of events (deaths at follow-up)	26	6	3	—

Mean ± Standard Deviation (SD) are presented for eGFR, age, serum creatinine. The *CKD5 group included N = 24 patients at ESRD (hence for these patients eGFR values were not available and the provided mean value is based on the remaining n = 9 CKD5 patients). **“Other than diabetes” refers to non-diabetic patients suffering from different renal diseases eg vascular and hypertensive nephropathy (15), Glomerular diseases (4), Interstitial nephropathy (4), Polycystic renal disease (3), Uninephrectoimy (4) [renal carcinoma (3) and infectious (1)], immunosuppressor toxicity (2), unknown (3) and normal (1).

**Figure 2:**
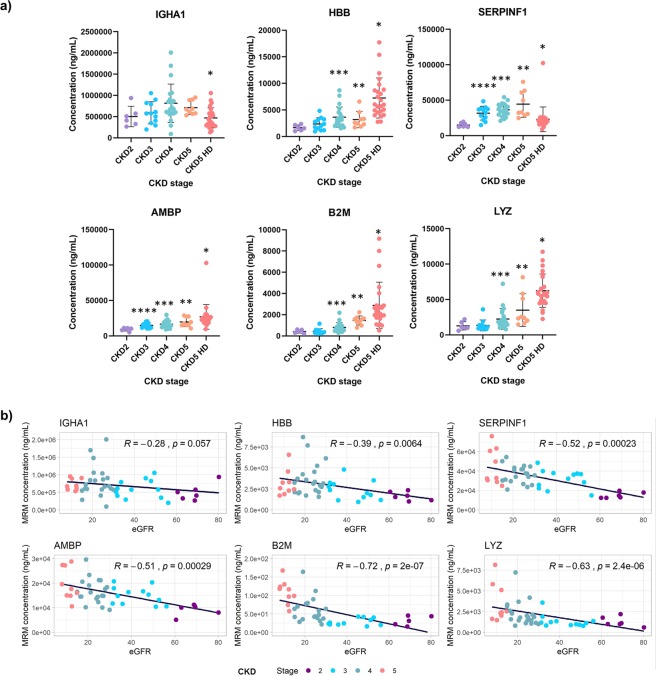
(**a**) Dot plot graph representation of the MRM quantification data for the six plasma proteins investigated. CKD5 is separated in two groups: (1) CKD5 patients (N = 9) and (2) CKD5 HD (hemodialysis patients, N = 24). *Significant differences of CKD5 HD patients with hemodialysis versus each of the other stages were observed for B2M, LYZ, AMBP, SERPINF1 and HBB with IGHA1 differing only in comparison to CKD 4. An impact of HD on the SERPINF1 levels may be observed with its levels decreasing significantly in comparison to CKD 5 patients. **Significant differences of CKD5 versus each of the other stages were observed for B2M, LYZ with AMBP, SERPINF1 and HBB differing only in comparison to CKD2. ***Significant changes could also be observed between CKD4 versus 3 (B2M, HBB) or CKD4 versus 2 (B2M, HBB, AMBP, LYZ, SERPINF1); ****Significant changes could also be seen for AMBP and SERPINF1 for CKD3 vs 2. (**b**) Correlation scores between eGFR and the MRM quantified proteins (ng/mL). Spearman correlation coefficients (R score) are shown.

Using the established assays, high quality spectra were obtained for all but one sample, where the data for SERPINF1, AMBP and B2M did not pass quality control (representative spectra of SIS and NAT peptides per marker are presented in Supplementary Fig. S1). Detailed information on MRM acquisition parameters and measurements per sample are provided in Supplementary Tables S4 and S5, respectively. The mean estimated abundance levels of the 6 proteins per CKD stage are shown in Fig. 2a. A significant change in CKD5 HD versus all other stages was observed for AMBP, B2M, LYZ, SERPINF1 and HBB (p < 0.05 based on Kruskal-Wallis test followed by Mann-Whitney pairwise comparisons), with B2M and LYZ also differing significantly when comparing CKD5 (excluding HD patients; n = 9) to the other stages. The lowest association with CKD stage was observed for IGHA1, found at increased levels only in CKD4 in comparison to CKD5 HD (p < 0.05).

As the investigated markers in the LC-MS/MS analysis by Glorieux *et al*.^37^ were compared between early (combined stages 2–3) and advanced CKD (stage 5) with HD, we performed the same comparison with our MRM data (Table 2). An overall agreement with the study by Glorieux *et al*.^37^ was observed, with most markers detected at increased abundance in CKD 5 with HD (N = 24) versus combined stages 2–3, except for IGHA1 and SERPINF1, where no significant changes could be detected (Table 2).

**Table 2:** Comparison of LC-MS/MS data^37^ [n = 15 patients of CKD5 with haemodialysis (HD) were compared to n = 14 patients with stage 2–3^37^] and LC-MRM-MS data [CKD stage 5 patients with HD (N = 24) versus CKD2-3].

Protein	Fold change CKD5 with HD/CKD2-3 in LC-MS/MS	Fold change of CKD5 with HD/CKD2-3 in LC-MRM-MS (Fold change CKD5 (all)/CKD2-3 in LC-MRM-MS)
IGHA1	1.88*	0.84 (0.95)
HBB	2.85*	3.41* (2.89*)
SERPINF1	1.92*	0.89 (1.11)
AMBP	3.98*	2.05* (1.90*)
B2M	99*	6.60* (5.71*)
LYZ	100*	4.71* (4.14*)

*Mann Whitney p < 0.05.

In addition, the fold change of n = 33 patients of CKD5 (including N = 24 with HD and N = 9 without HD) versus n = 18 patients with CKD2-3 is shown in parenthesis.

B2M, followed by LYZ plasma concentrations showed strong negative correlations to eGFR levels (R = −0.72 and −0.63, respectively) (Fig. 2b). For this analysis, 24 CKD stage 5 patients on HD were excluded due to lack of baseline eGFR values (Supplementary Table S3). Negative correlations were also observed for AMBP, SERPINF1 and HBB with the weakest being observed for the latter (HBB), whereas for IGHA1 significance was not reached (p = 0.057) (Fig. 2b).

### Correlation of MRM measurements to ELISA

**Figure 3:**
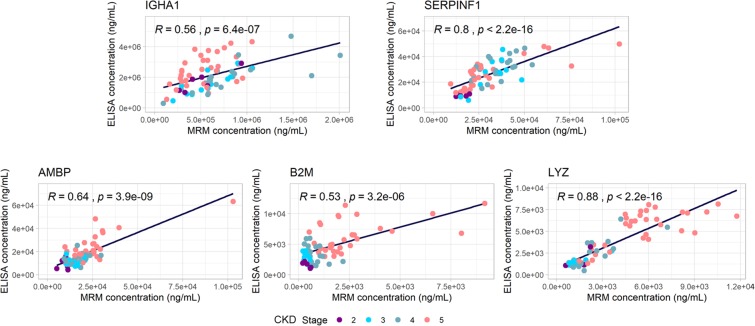
Spearman correlation analysis of MRM and ELISA data in the same sample cohort.

To further validate the MRM measurements, ELISAs were performed for 5 out of the 6 proteins where specific and well characterized assays were available (IGHA1, SERPINF1, AMBP, B2M, LYZ). Indeed, the correlation of the findings of the 2 methods was to the least moderate (IGHA1 and B2M with Rho = 0.56 and Rho = 0.53, respectively) or in most cases high (for proteins LYZ, SERPINF1 and AMBP with Rho = 0.88, Rho = 0.8 and Rho = 0.64 respectively; Fig. 3) further supporting the validity of the MRM results **(**Fig. 3**)**. ELISA measurements per sample are presented in Supplementary Table S6.

### Association to outcome

**Figure 4:**
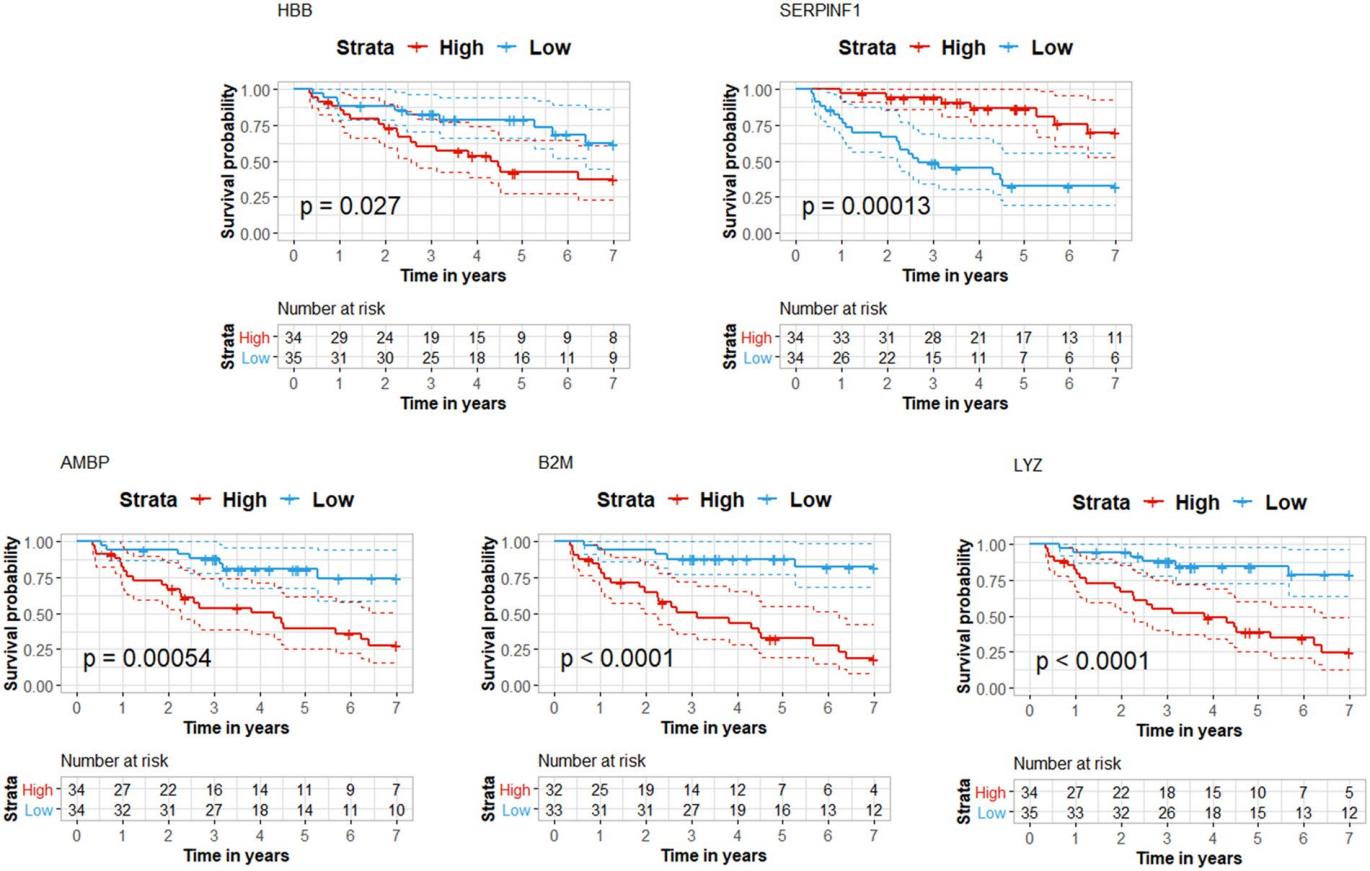
Survival analysis for MRM quantified proteins. Kaplan-Meier plots depicting 7 years’ survival probability for patients with CKD stratified based on median protein concentration (ng/mL) as defined by MRM. Dashed lines indicate confidence intervals at 95% level. Significant differences in survival (p < 0.05) are determined with the Log-rank test.

To investigate association of the quantified markers to outcome (death), Kaplan Meier analysis was performed. Given its overall poor associations to staging as well as lack of correlation to eGFR, IGHA1 was excluded from further analysis. As shown in Fig. 4, higher (based on a median cut-off) plasma concentrations (ng/mL) of HBB (p = 0.027), AMBP (p = 0.00054), B2M (p < 0.0001) and LYZ (p < 0.0001) and lower plasma concentrations of SERPINF1 (p = 0.00013) were significantly associated with death.

### Establishment of classifier

**Figure 5:**
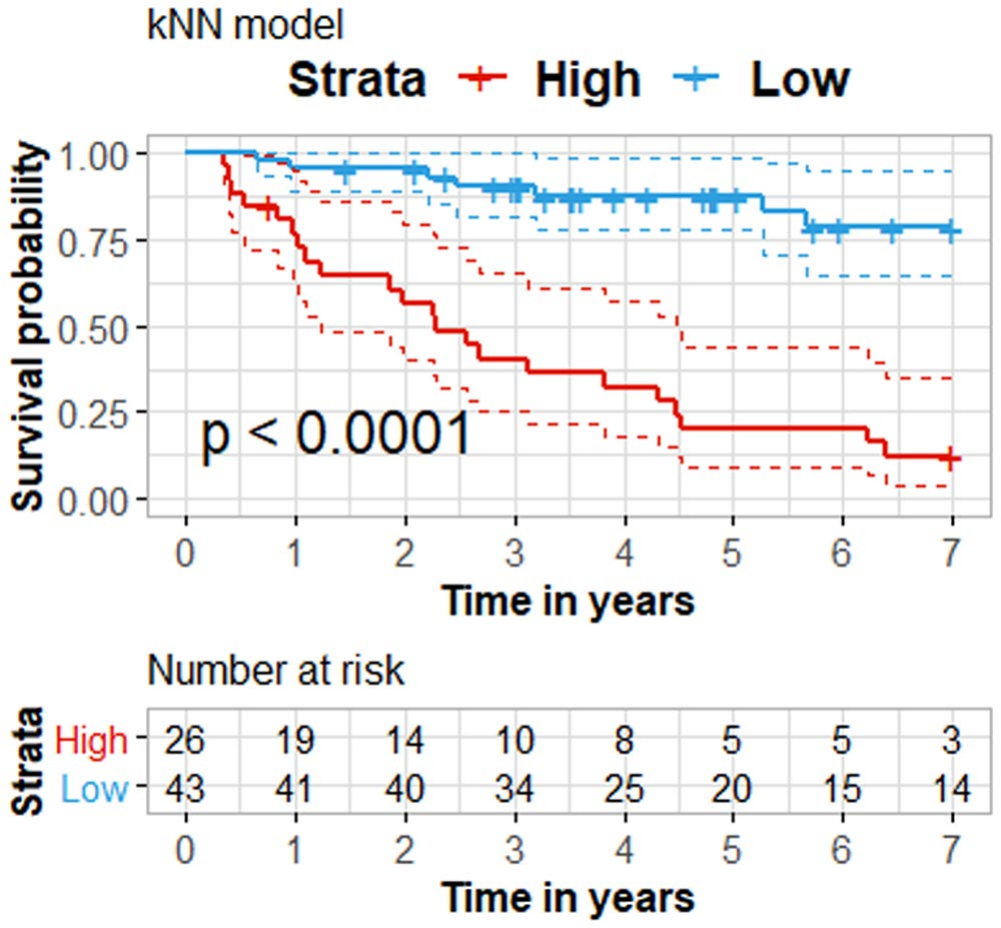
Survival analysis of high vs low risk groups predicted with k-nearest neighbor classifier. The classifier was trained to distinguish between censored and deceased data from patients with CKD, using leave-one-out crossvalidation.

To assess the added value of combining the measured markers into a single simple classifier we utilized instance-based learning. In that, a k-nearest neighbor (knn) classifier was fed with the MRM data and was trained to distinguish between disease status at 7 years follow up time. The model was validated in predicting risk for mortality by leave one out crossvalidation. As the levels of SERPINF1 were found to be highly affected by HD (Fig. 2a), the marker was excluded and the knn model was developed based on the concentrations (ng/mL) of the remaining 4 proteins, HBB, AMPB, B2M and LYZ. At 7 years’ follow up, using leave-one-out crossvalidation, subjects were predicted either as “deceased” (high risk group) or “censored” (low risk group). Kaplan – Meier analysis of the predicted high/low risk groups illustrated significantly shorter survival time for the high risk group (Fig. 5), suggesting that the combination of HBB, AMBP, B2M and LYZ may efficiently predict survival for patients with CKD.

**Figure 6:**
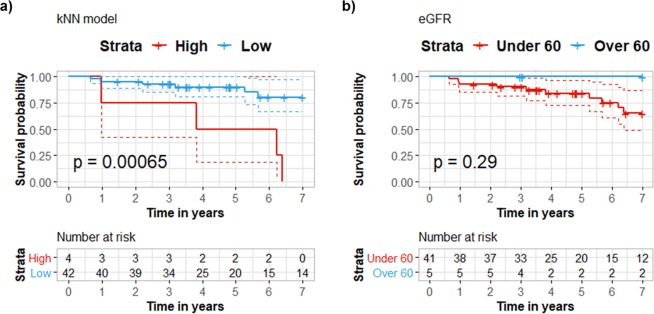
Comparison of survival probability based on a sub-cohort of 46 patients where eGFR measurements are available. (**a**) Survival analysis in the same group of patients of 46 patients of high vs low risk groups predicted with k-nearest neighbor classifier. The classifier was trained to distinguish between censored and deceased data from patients with CKD, using leave-one-out crossvalidation. (**b**) Survival probability is evaluated based on eGFR measurements under and over 60 mL/min/1.73 m^2^. Based on the p value, the KNN classifier seems to predict more effectively the occurrence of death compared to eGFR.

Furthermore, a similar analysis was performed in 46 patients with available eGFR measurements in plasma, as eGFR is classically used for the evaluation of CKD progression (Fig. 6). The classifier model suggested that the combination of HBB, AMBP, B2M and LYZ may predict more efficiently survival for patients with CKD (p < 0.05) (Fig. 6a) compared to eGFR measurements (p > 0.05), when the cutoff of eGFR was set at 60 mL/min/1.73 m^2^ (Fig. 6b).

## Discussion

Multiple biomarkers for CKD progression towards ESRD and death, widely classified as biomarkers of kidney function and kidney damage, have been described^17,54–57^. eGFR and albuminuria are the main clinically used markers, despite the extensively described shortcomings in terms of diagnostic and prognostic accuracies^17^. Multiple additional proteins, including various tubular markers (such as kidney injury molecule-1, neutrophil gelatinase-associated lipocalin, cystatin C, a-1-microglobulin) have been described in association with the disease^17,54,58^ but generally are not routinely implemented, yet.

To address disease complexity, multi-parametric, high dimensional classifiers incorporating numerous disease-specific and systemic molecular changes are increasingly being established, as better reflecting the disease molecular heterogeneity^59^. A prominent example is the multi-peptide urinary classifier CKD273 being used for the detection of nephropathy at early stages^26,29^.

We employed a multiplex MRM mass spectrometry assay for the quantification of six previously described, exploratory CKD markers. MRM, in contrast to the classically employed highly sensitive ELISA assays, does not require the use of specific antibodies, thus facilitating multiplexing and increasing specificity via eliminating antibody cross-reactivity problems^32,33^. MRM has been widely applied in plasma for proteomic biomarker validation^60^ and for various plasma proteins (such as apolipoproteins). It has been demonstrated to provide assay performance equal to well characterized ELISA assays^61^. In fact, the observed correlations between our MRM data and respective independent immunoassays (Rho = 0.53–0.88) are in line to existing literature and reported acceptable correlations of such assays^62,63^.

Besides evidence of association with CKD, the selection of the specific markers was driven by the existence of mass spectrometry-based data per protein to ensure detectability, as well as expected abundance levels to eliminate the need for extensive pre-fractionation. The detection limits observed in our study (in the range of ng/mL) are similar to ones regularly reported when using unfractionated plasma^64^. Applications involving combination of MRM with immunoprecipitation protocols^62^ or peptide separation by two dimensional liquid chromatography can extent the quantification range from below 100 ng/mL to about 500 pg/mL range^65^. However, these protocols are substantially more elaborate, and not well suited for routine implementation.

Among the analyzed proteins, B2M is the most well-characterized CKD-associated marker and known uremic solute according to the European Uremic Toxin Work Group^66^. It is a component of the class I major histocompatibility complex (MHC), synthesized normally by lymphocytes, filtered in the glomerulus and catabolized by the proximal tubular cells^67,68^. Upon defective renal function, its serum levels increase significantly^66–68^. In line with our study, gradual increase in B2M levels across stages of CKD has been shown^38^. Furthermore, several associations between B2M levels and death, dialysis or cardiovascular events have been reported^39,42,43^, in agreement with our findings.

Pigment epithelium-derived factor (SERPINF1), initially characterized as a neuronal differentiation factor in retinoblastoma cells^69^, was later defined as a member of the serine protease inhibitor family with antiangiogenic, antioxidative, anti-inflammatory, and antitumorigenic activity^70^, and was recently assigned a renoprotective role^71^. In line with our analysis, SERPINF1 serum levels were found elevated in ESRD patients compared to healthy controls^47^. Similarly, SERPINF1 was reported increased in plasma of CKD3 patients in comparison to controls^48^. In addition, Hui *et al*., demonstrated that SERPINF1 levels correlated with eGFR (n = 1136), and further associations to CKD progression (n = 1,071 stages 1–3 of which 171 progressed) were supported^49^. In our study, surprisingly, an association of lower SERPINF1 levels with death was detected. This finding is most likely driven by the HD patients included in the cohort. In fact, if we exclude HD patients from the analysis, higher levels of the protein in CKD5 vs CKD4 can be observed (Fig. 2a).

AMBP has been studied mainly in urine and to a lesser extent in plasma and tissue in the context of CKD. Increased urinary AMBP levels have been associated with kidney damage^72^ and tubular dysfunction in diabetic nephropathy^73^. In addition, several shotgun proteomic studies followed by verification via different assays (Western blot, MRM in small sample sizes (n ≤ 11), or ELISA), supported an upregulation of AMPB in patients of various CKD stages compared to healthy controls, in adipose tissue, urine as well as urine exosomes^74–76^. AMBP was also found to progressively increase in plasma of individuals with increasing CKD stage –from CKD1-2 to CKD3-4 and finally to CKD5- based on relative quantification by LC-MS/MS^77,78^ or MRM^77^.

AMBP, SERPINF1, B2M, and HBB have been investigated as HDL (High-density lipoprotein) associated proteins by LC-MS/MS, and were found to correlate with severe kidney damage^79^. This latter study also revealed elevated levels of these proteins in ESRD patients and recipients with poor graft function compared to patients with good graft function, a finding which was further confirmed by Western Blot analysis for SERPINF1 and AMBP^79^. Our study also supports the progressive up-regulation of AMPB in plasma with increasing CKD stage, negative correlation to eGFR and unfavorable outcome (p = 0.00054).

In comparison to B2M, SERPINF1 and AMBP, the published evidence associating IGHA1, HBB and LYZ to CKD is limited. IGHA1 was found at higher levels in the plasma of patients with CKD5 with HD in comparison to CKD2-3 in the study by Glorieux *et al*.^37^. This finding could not be verified in our study, which also failed to demonstrate association of the protein to eGFR. In contrast, the differential abundance of HBB, one of the two polypeptide chains that form Hemoglobin A^80^, in CKD5 versus earlier stages was verified and a modest association to eGFR was observed. The most pronounced finding of our study is the clear associations of LYZ with all CKD stages, eGFR as well as survival. LYZ is released from leukocytes and macrophages, and has antibacterial properties and an attributed role in myocardial depression and vasodilation^81^. With the exception of the study by Glorieux *et al*.^37^, and an *in vitro* study suggesting anti-inflammatory properties of LYZ on human proximal tubular epithelial cells (HK-2 cells)^82^, studies of LYZ in the context of CKD are lacking. Based on our results, such studies seem well justified, which may also expand to the investigation of cardiorenal syndrome, considering reports associating plasma levels of LYZ to coronary artery disease severity^82,83^.

Collectively, our study describes a multiplex assay for the absolute quantification and verification analysis of previously described putative markers for CKD, laying the groundwork for further investigation in prospective validation studies. As shown, the above markers seem to correlate with CKD stage (Fig. 2a). An extra comparison of protein concentrations between pathological conditions other than CKD (diabetic vs non diabetic as well as hypertensive vs non hypertensive patients, as per availability) was performed for the 6 studied proteins. The overall lack of statistical significance (MW, p > 0.05) in these cases (Supplementary Fig. S2**)** suggests that our targets could be specific markers for CKD progression. However, the power of the study is too small and further validation is needed to confirm this observation. Overall, the small sample size and restriction to 6 markers are clear limitations. Furthermore, larger scale studies will be required to validate changes per stage including in early disease. The small sample size does not also allow multivariate analysis to be conducted with confidence. An effort to investigate the added value of the classifier on top of clinical variables was performed and even though an independent prognostic value over age could be seen, this was lost with the addition of CKD stage (the output of cox proportional hazard model is summarized in Supplementary Table S7). Nevertheless, the availability of follow-up information allowing preliminary associations to outcome and simplicity of the assay, avoiding large fractionation schemes, provide a strong basis prompting further advancement towards properly sized validation trials.

## Methods

### Study population

The study population consisted of all 72 patients (outpatient and dialysis unit of Nephrologie Dialyse Saint Guilhem and the Public Hospital of Sète, as well as the Department of Nephrology, Transplantation and Dialysis of the University Hospital of Montpellier) that were recruited for the “Urosysteomics” study^26^. Of these, 24 were classified as ESRD patients at baseline. The study was approved by the Comité de Protection des Personnes of Montpellier and declared to the French Ministry of Health (reference number DC-2008–417). All methods were performed in accordance with the relevant guidelines and regulations. All individuals gave written informed consent. Pertinent clinical data are provided in Supplementary Table S3. Estimated glomerular filtration rate (eGFR) was calculated using the CKD-EPI equation^84^.

### Selection of peptides for LC-MRM-MS

For the establishment of the MRM assays, proteotypic peptides were selected considering the following criteria: (i) the peptides uniquely represented the target protein, (ii) lysine or arginine was allowed only at the carboxy terminus (no missed tryptic cleavage sites are allowed), (iii) peptide sequence included 6–25 amino acid residues to ensure acceptable ionization and gas-phase fragmentation, and (iv) amino acids that are susceptible to chemical modifications such as cysteine, methionine should be absent. To perform the selection, multiple tools were employed in combination: a spectral library from the National Institute of Standards and Technology (NIST) [“human consensus final true lib”, downloaded from http://www.nist.gov/] on 05062014] was imported into Skyline software. The Skyline indicated proteotypic peptides and respective transitions per peptide which were further validated with additional bioinformatics tools: The PeptidePicker (http://mrmpeptidepicker.proteincentre.com/peptidepicker9/) that predicts proteotypic peptides with similar criteria as Skyline^85^; The Peptide Tracker (http://tracker.proteincentre.com/) that records transitions and chromatographic properties of specific proteotypic peptides based on previously performed MRM assays^86^, and the CPTAC Assay Portal (https://assays.cancer.gov/available_assays) that compiles information of existing MRM assays (proteotypic peptides, analytical performance, suggested protocols)^87^. In addition, shortlisted peptides were evaluated using the Protein Basic Local Alignment Search Tool (BLAST, http://blast.ncbi.nlm.nih.gov) to ensure their proteotypicity.

For the peptides identified (4–7 per biomarker) and their respective transitions (3–5 per peptide), based on the aforementioned procedure and considered for further analysis, initial MRM experiments were conducted using two plasma samples (normal and CKD, respectively) in triplicate. Further shortlisting was performed based on: (i) the quality of the MS/MS spectrum of each peptide in the human spectral library, (ii) score and number of observations in MS-based proteomics experiments, as provided from PeptideAtlas (http://www.peptideatlas.org)^88^ and (iii) quality and reproducibility of the MS/MS spectrum of the native (NAT) peptide in the two tested plasma samples as defined by: (a) peptide transitions should co-elute and yield Gaussian peak shapes with insignificant fronting and tailing and (b) the same elution profiles should have been observed in the spectral library. Based on these criteria, one peptide per biomarker and 2–5 transitions per peptide were selected for quantification in the clinical samples. A stable isotope-labeled version of each peptide, was synthesized through incorporation of ^13^C and ^15^N isotopes into Lysine (K) or Arginine (R) C-terminal residues, for use as internal standards [stable isotope standard (SIS) peptide] (Thermo Scientific, JPT). As the employed labeled peptides were not of highest purity, their purity and final concentrations were defined by MS analysis (MALDI TOF MS for AMBP, HBB, SERPINF1, IGHA1), parallel reaction monitoring (PRM) in comparison to standard/recombinant protein for B2M, or estimation of the labeled peptide amount by correlating 4 different analytical runs (not considered for the definition of the presented clinical associations) to respective ELISAs for LYZ.

### Sample preparation and liquid chromatography-multiple reaction monitoring-mass spectrometry (LC-MRM-MS)

Equal volume (2 μL) of plasma samples containing approximately 100 *μ*g of total protein were used for LC-MRM-MS analysis as previously described^89,90^. Briefly, after protein denaturation (8 M urea), reduction (10 mM dithioerythritol) and alkylation (50 mM iodoacetamide) the samples were digested with trypsin [(1:100 enzyme: protein ratio (w/w)] for 16 hours in the dark (RT). The peptide mixture was desalted with solid phase extraction zip-tips (Thermo Scientific) and the extracted peptides were dried using a vacuum centrifuge. The dried peptides were solubilized in mobile phase A (97.9% H_2_O, 2% acetonitrile, 0.1% formic acid), pH 3.5 to obtain a final concentration of 0.5 *μ*g/*μ*L. A mixture of the SIS peptides was then added in each sample after drying the peptides as follows: IGHA1: 8000 ng/mL, B2M: 800 ng/mL, HBB: 770 ng/mL, AMBP: 2240 ng/mL, LYZ: 800 ng/mL, SERPINF1: 668 ng/mL. Liquid chromatography was performed using an Eksigent nano-HPLC system, coupled with a C18 analytical column (75 μm × 150 mm, particle size 5 μm, pore size 100 Å) (Thermo Scientific). Peptide separation and elution were performed with a 60 min gradient of 5–90% mobile phase B (80% acetonitrile v/v, 0.1% FA, 19.9% H_2_O) at a flow rate of 300 nL/min. Samples were injected into the LC system and loaded on the C18 column. Tryptic peptides were analyzed on an AB Sciex 4000QTRAP with a nanoelectrospray ionization source controlled by Analyst 1.5 software (AB Sciex). The mass spectrometer was operated in MRM mode, with the first (Q1) and third quadrupole (Q3) at 0.7 unit mass resolution. Detailed information about the acquisition method and the used parameters are provided in Supplementary Table S4.

### Standard curve

To define the range and ensure linearity in measurements, a calibration curve for each peptide was generated spiking SIS peptide at different amounts in a CKD pooled plasma sample (CKD stages 2–5, n = 4), in order to maintain the same background matrix to the analyzed samples. Dilution points were selected to cover reported concentration ranges per marker based on the literature and/or reported MS data and each dilution point was analyzed in triplicate. Samples were run in LC-MRM-MS, as described above.

### Data analysis and quantification

Data analysis was performed using the Skyline software^91^ and all chromatograms were manually inspected to ensure the good quality and accurate peak picking. In particular, manual inspection ensured that the extracted ion chromatograms of the stable isotope-labeled standard (SIS) and endogenous (or natural-NAT) peptides chromatographically co-eluted and exhibited identical peak symmetry, shape, and width. The top signal producing transition was selected as the quantifier transition in all cases, while the remaining transitions were used as qualifier transitions, for accurate peak profile and retention time confirmation. In the case of standard curve, linear regression analysis was performed to define the slope and linearity of measurements.

Finally, the NAT/SIS ratio [Light/Heavy-relative response (RR)] of the quantifier transition was used for quantification (peak area of quantifier transition in NAT peptide/peak area of quantifier transition in SIS peptide). Concentrations were reported to ng/mL by using the protein molecular weights, as determined by Uniprot (https://www.uniprot.org).

### ELISA

The same cohort of samples (N = 72) was analyzed with ELISA to investigate the correlation among the two different methods. The ELISA kits that were employed, specifically, included: for protein bikunin/AMBP: DY7744-05/R and D systems;, for SERPINF1: ab213815/Abcam, LYZ: ab108880/Abcam, IGHA1: ab196263/Abcam, and B2M: ab108885/Abcam. Values could not be obtained for n = 1 samples for SERPINF1 and n = 3 samples for B2M.

### Statistical analysis (correlation-survival analysis)

Visualization of the stage analysis was performed by GraphPad Prism version 8.0.0 for Windows (GraphPad Software, San Diego, California USA, www.graphpad.com), whereas statistical and survival analysis were conducted in the R (version 3.6.1) environment for Windows utilizing base functions from the packages stats, survival and survminer. Significant changes at the levels of continuous variables across groups were determined with the non-parametric Kruskal-Wallis and Mann-Whitney tests. Kaplan-Meier plots were constructed to compare survival between Low and High protein concentration (ng/mL) groups, defined by a median cut-off point per protein, across samples. Survival curves were compared for significant differences (p < 0.05), from baseline diagnosis to 7 years follow-up time with the Log-rank test. Similarly, correlation analysis between eGFR and MRM quantifications were performed. Linear relationships between the continuous variables were assessed using Spearman’s Rank correlation coefficient and significance was defined at p ≤ 0.05.

### Predictive value of the MRM panel

The prognostic value of the quantified proteins was assessed in distinguishing disease status at 7 years follow up time. The k-nearest neighbor classifier was developed with the packages DMwR, and pROC, in the R environment for Windows. In particular, the complete MRM data of 4 proteins (HBB, AMBP, B2M and LYZ) quantified across 69 subjects with CKD were utilized to build the model and parameter optimization was assessed in a 3-fold cross validation setting. In brief, patients were divided into three random partitions (adjusted for a balanced ratio of deceased/censored, stage and class cases), each time using two out of the three partitions for training, and the remaining third as a test set. Summary statistics of the classification performance for the classifier were obtained for the three folds and optimization for most suitable number of k-nearest neighbors was conducted iteratively as a function of maximizing the Area Under the Receiver Operating Characteristic curve across folds. Best fit was recorded for k = 16 neighbors and this number was further utilized to establish the final leave-one-out-crossvalidation (loocv) model. Patient specific probability scores of being labeled as deceased (scores ranging from 0.5 to 1) or censored (scores ranging from 0 to 0.5) were obtained with loocv and were converted to binary groups of “high” and “low risk”, respectively. Significant differences in survival between high and low risk groups were investigated with the Log-rank method, as described. Missing values (1.71% of the data) were replaced with the limit of quantitation for each protein (minimum concentration). Data for the knn classification were subjected to log transformation prior to training. The cox proportional hazard model was developed with the packages survival, survminer and fitting of proportionality was evaluated and examined with the functions *cox.zph* and *ggcoxzph*, respectively.

## Supplementary information

Supplementary File.

Supplementary Table S1.

Supplementary Table S2.

Supplementary Table S3.

Supplementary Table S4.

Supplementary Table S5.

Supplementary Table S6.

Supplementary Table S7.

## Data Availability

All data generated or analyzed during this study are included in this published article (and its Supplementary Information Files). As described above, all methods were carried out in accordance with relevant guidelines and regulations. Skyline data are available upon request.
