# Supplementary material for: Multiplexed MRM-based protein quantification of putative prognostic biomarkers for chronic kidney disease progression in plasma
Source: Sci Rep. 2020 Mar 16;10:4815. doi: 10.1038/s41598-020-61496-z (PMC7076027; doi:10.1038/s41598-020-61496-z)
Supplement: Supplementary file 1 — Supplementary File. [file 41598_2020_61496_MOESM1_ESM.docx]

**Multiplexed MRM-based protein quantification of putative prognostic biomarkers for chronic kidney disease progression in plasma**

*Supplementary Data*

Manousos Makridakis^+1^, Georgia Kontostathi^+1^, Eleni Petra^1^, Rafael Stroggilos^1^, Vasiliki Lygirou^1^, Szymon Filip^1^, Flore Duranton^2^, Harald Mischak^3^ , Angel Argiles^2^, Jerome Zoidakis^1^, Antonia Vlahou^1^*

^+^equal contribution

*corresponding author

^1^Biotechnology Division, Biomedical Research Foundation, Academy of Athens (BRFAA), Athens, Greece

^2^RD-Néphrologie, Montpellier, France

^3^Mosaiques Diagnostics, Hannover, Germany

Supplementary Figure S1


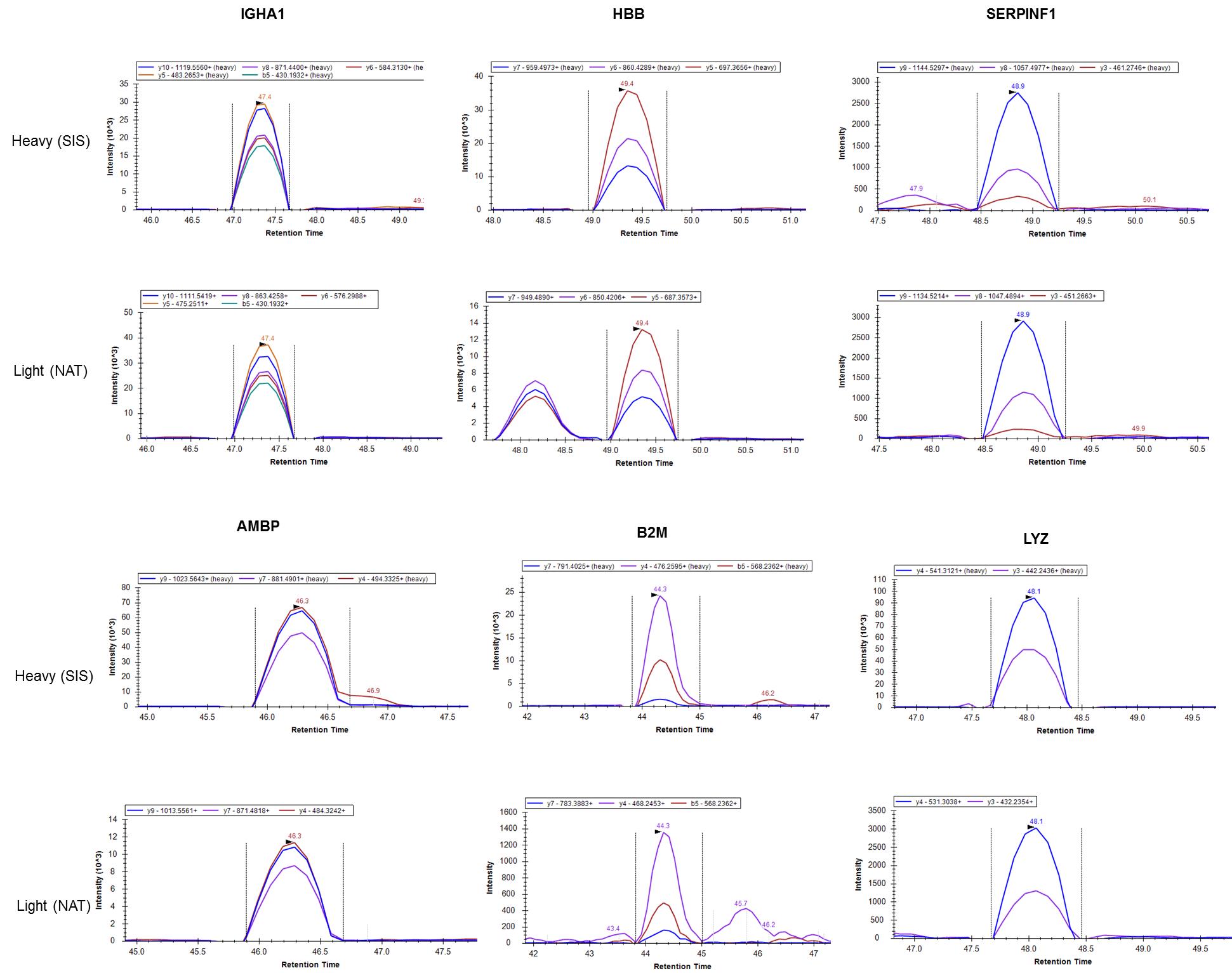


Supplementary Figure S2


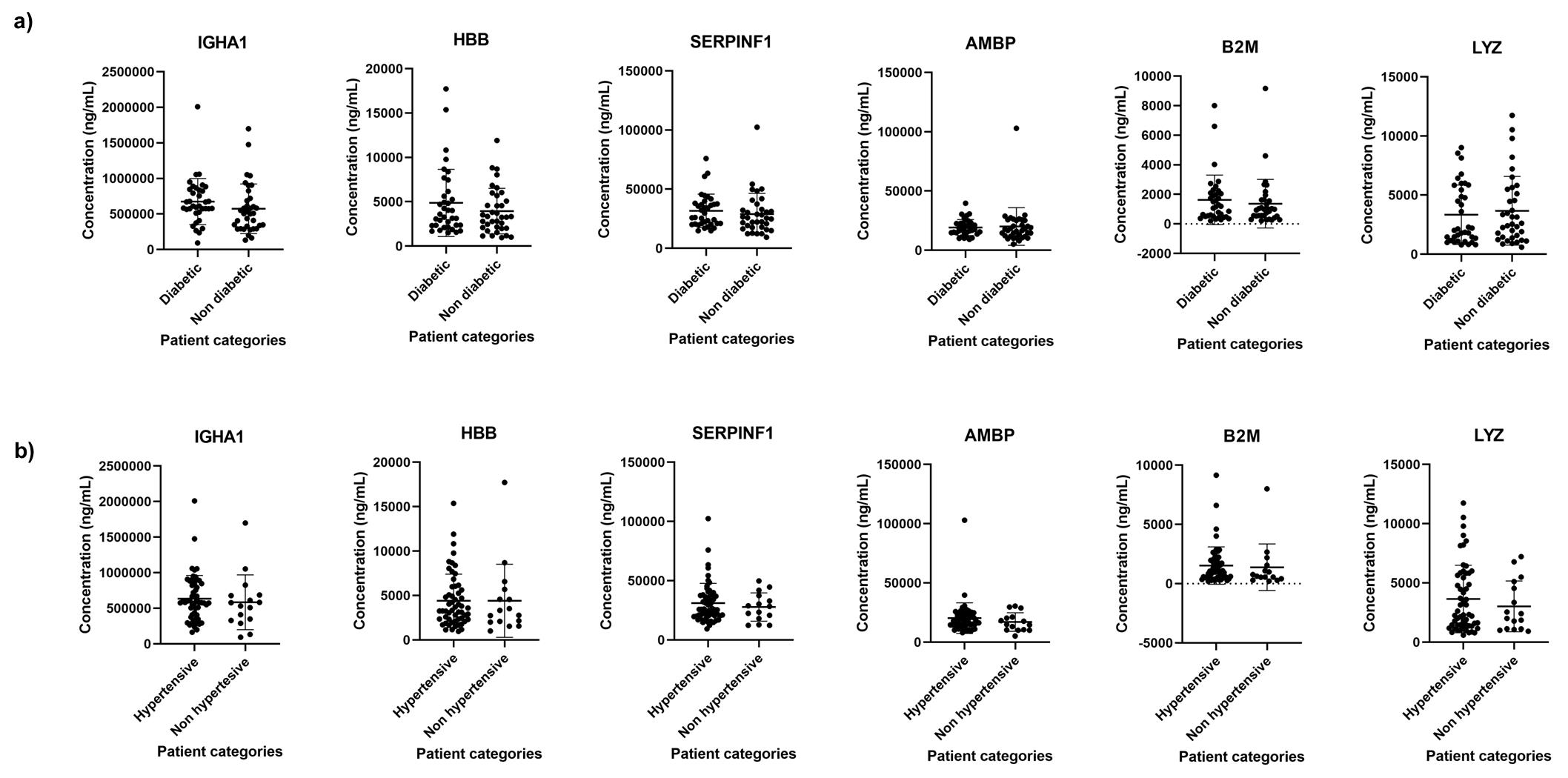


**Supplementary Tables Legends**

Supplementary Table S1: Reference values (ng/mL) in normal plasma or less often in CKD patients.

Supplementary Table S2: Detailed analytical characteristics of standard curves for IGHA1, HBB, SERPINF1, AMBP, B2M and LYZ.

Supplementary Table S3: Clinical info of the Montpellier cohort.

Supplementary Table S4: Specific MRM acquisition parameters for the 6 target peptides (corresponding to 6 proteins) monitored by LC-MRM-MS. The quantifier transition that was used for quantification is highlighted in yellow fill colour, whereas the rest transitions served as qualifiers for accurate peak picking.

Supplementary Table S5: Detailed absolute quantification data for the measured samples per protein via MRM.

Supplementary Table S6: Correlations of MRM-ELISA results

Supplementary Table S7: Output of cox proportional hazard model.

**Supplementary Figure Legends**

Supplementary Figure S1: Extracted ion chromatograms (XICs) of transitions monitored heavy (SIS) and endogenous (NAT) proteotypic peptides for each protein. The SIS peptide co-elutes with its corresponding NAT peptide and their transitions (fragments) exhibit identical peak symmetry, shape and width.

Supplementary Figure S2: Dot plot graph representation of the MRM quantification data for the six plasma proteins investigated, where the following comparisons were performed: a) Diabetic versus non diabetic patients and b) Hypertensive vs no hypertensive patients. There were no significant differences (MW, p>0.05) in either of the above comparisons (a, b) in any of the studied proteins (IGHA1, HBB, SERPINF1, AMBP, B2M, LYZ).
